# *Ilex latifolia* Thunb protects mice from HFD-induced body weight gain

**DOI:** 10.1038/s41598-017-15292-x

**Published:** 2017-11-07

**Authors:** Hailan Wu, Yue-Lei Chen, Yueyuan Yu, Jin Zang, Yikuan Wu, Zhao He

**Affiliations:** 1 State Key Laboratory of Food Science and Technology, School of Food Science and Technology, Jiangnan University, Wuxi, 214122 China; 2 Synergistic Innovation Center for Food Safety and Nutrition, School of Food Science and Technology, Jiangnan University, 1800 Lihu Avenue, Wuxi, Jiangsu 214122 China; 3 Institute of Endocrinology and Metabolism, Shandong Academy of Clinical Medicine, Shandong Provincial Hospital affiliated to Shandong University, Jinan, Shandong 250021 China; 4 Institute of Biochemistry and Cell Biology, Shanghai Institutes for Biological Sciences, Chinese Academy of Sciences, Shanghai, 200031 China

## Abstract

Kuding tea is implicated in alleviating metabolic disorders in traditional Chinese medicine. However, the role of *Ilex latifolia* Thunb (kuding tea), one of the large leaf kuding tea species, in the prevention of the development of obesity remains to be determined. We show here that 7-week-old male mice treated with an *Ilex latifolia* Thunb supplement for 14 weeks were resistant to HFD-induced body weight gain and hepatic steatosis, accompanied by improved insulin sensitivity. *Ilex latifolia* Thunb supplementation dramatically reduced the systemic and tissue inflammation levels of mice via reducing pro-inflammatory cytokine levels, increasing anti-inflammatory cytokine levels in the circulation and inhibiting p38 MAPK and p65 NF-κB signaling in adipose tissue. Together, these results indicate that *Ilex latifolia* Thunb protects mice from the development of obesity and is a potential compound pool for the development of novel anti-obesity drugs.

## Introduction

Green tea and kuding tea are two of the most popular beverages in China^1,2^. Green tea has been well studied for its health benefits in previous reports, but the biological functions of kuding tea are not as well studied^3–5^. The kuding teas most commonly found in the markets of China are typically classified into two groups: one group, which includes at least two species, *Ilex latifolia* Thunb and *Ilex kudingcha* C.J. Tseng (*Ilex kaushue*), has been well characterized as “large-leaved Kudingcha”; the other group, consisting of the species *Ligustrum robustum*, has been classified as “small-leaved Kudingcha”^6^. Kuding tea has been widely used in Chinese medicine for more than 2,000 years as a beverage to quench thirst, remove phlegm, refresh the mind and improve eyesight^6,7^. Previous studies have investigated pharmaceutical functions of *Ilex kudingcha* C.J. Tseng, including anti-oxidant, anti-hypertensive, anti-obesity, anti-diabetic, hepatoprotective and anti-cancer roles^8–10^. However, experimental data on the effects of *Ilex latifolia* Thunb on health are lacking.

Here, we found that aqueous extract of *Ilex latifolia* Thunb inhibited lipogenesis via suppressing the expression of lipogenic genes. Aqueous extract of *Ilex latifolia* Thunb prevented body weight gain and hepatic steatosis, enhanced insulin sensitivity and reduced chronic inflammation in mice. The alleviation of body weight gain by *Ilex latifolia* Thunb suggested that herbal supplements of *Ilex latifolia* Thunb could be used in the intervention of development of obesity.

## Results

### Aqueous extract of *Ilex latifolia* Thunb decreases adipocyte lipid accumulation

**Figure 1:**
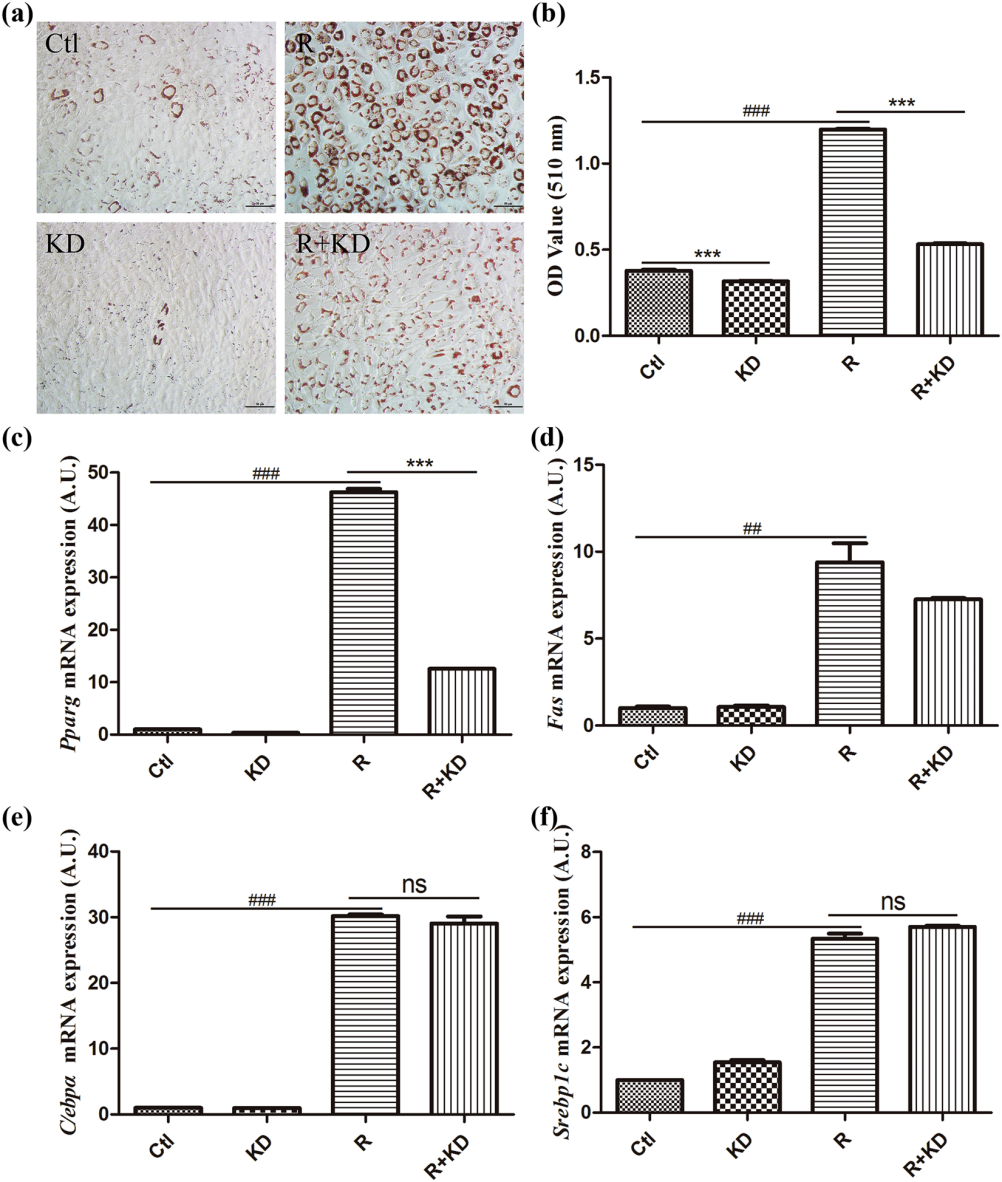
Adipose differentiation in OP9 mouse stromal cells. (**a**) The cells were stained with oil red O at day 5. (**b**) The absorbance was measured at 510 nm. (**c–f**) qPCR was used to determine mRNA levels in OP9 mouse stromal cells. The aqueous extract of *Ilex latifolia* Thunb was added to the medium at 4 μg/mL. PBS was used as the vehicle control. *Pparg: Ppar*_*γ*_; Ctl: control; R: rosiglitazone; KD: aqueous extract of *Ilex latifolia* Thunb. *β-actin* was used as an internal control. Data are presented as the mean ± SEM. Significant differences between Ctl and R are indicated as ^##^P < 0.01, ^###^P < 0.001; significant difference versus R + KD or KD are indicated as ***P < 0.001.

*Ilex kudingcha* C.J. Tseng (kuding tea) has been implicated in the prevention of obesity and diabetes in traditional Chinese medicine^1,11^. To investigate the function of *Ilex latifolia* Thunb on obesity development, we first examined the effects of the aqueous extract of *Ilex latifolia* Thunb on the prevention of adipocyte differentiation of OP9 mouse stromal cells. Rosiglitazone was used as an inducer to promote the adipocyte differentiation of OP9 cells, and then an aqueous extract of *Ilex latifolia* Thunb was added into the medium from day 1 to day 5. Compared with the control, *Ilex latifolia* Thunb significantly decreased lipid accumulation, indicating an inhibitory effect of *Ilex latifolia* Thunb on lipid accumulation (Fig. 1a,b). Moreover, the aqueous extract of *Ilex latifolia* Thunb displayed significantly stronger inhibitory effects on OP9 cell lipogenesis than the aqueous extract of *Ilex kudingcha* C.J. Tseng (Fig. S1).

Previous studies have shown that *Ppar*_*γ*_, *C/ebpα*, *Srebp1c* and *Fas* are critical genes in the regulation of adipogenesis and accumulation of fatty acids^12–14^. To verify whether their expressions were altered by *Ilex latifolia* Thunb, we first examined the mRNA levels of *Ppar* *γ*, *C/ebpα*, *C/ebpβ*, *Adiponectin*, *Srebp1c* and *Fas* in cells by a quantitative PCR approach. We found that the mRNA levels of *Ppar*_*γ*_ were decreased in *Ilex latifolia* Thunb-treated cells compared to control cells, indicating that *Ilex latifolia* Thunb inhibits lipid accumulation via *Ppar* *γ* signaling (Fig. 1c). However, no change in *C/ebpα*, *C/ebpβ* and *Srebp1c* mRNA levels was observed in *Ilex latifolia* Thunb-treated cells compared to control cells, suggesting that *C/ebpα* or *Srebp1c* signaling is not involved in the inhibition of lipogenesis by *Ilex latifolia* Thunb (Figs 1d–f and S2b). The treatment of aqueous extract of *Ilex latifolia* Thunb increased *Adiponectin* mRNA levels in OP9 cells (Fig. S2a). Taken together, these results suggest that *Ilex latifolia* Thunb inhibits lipid accumulation due to suppression of *Ppar* *γ* transcription.

### *Ilex latifolia* Thunb alleviates HFD-induced body weight gain

**Figure 2:**
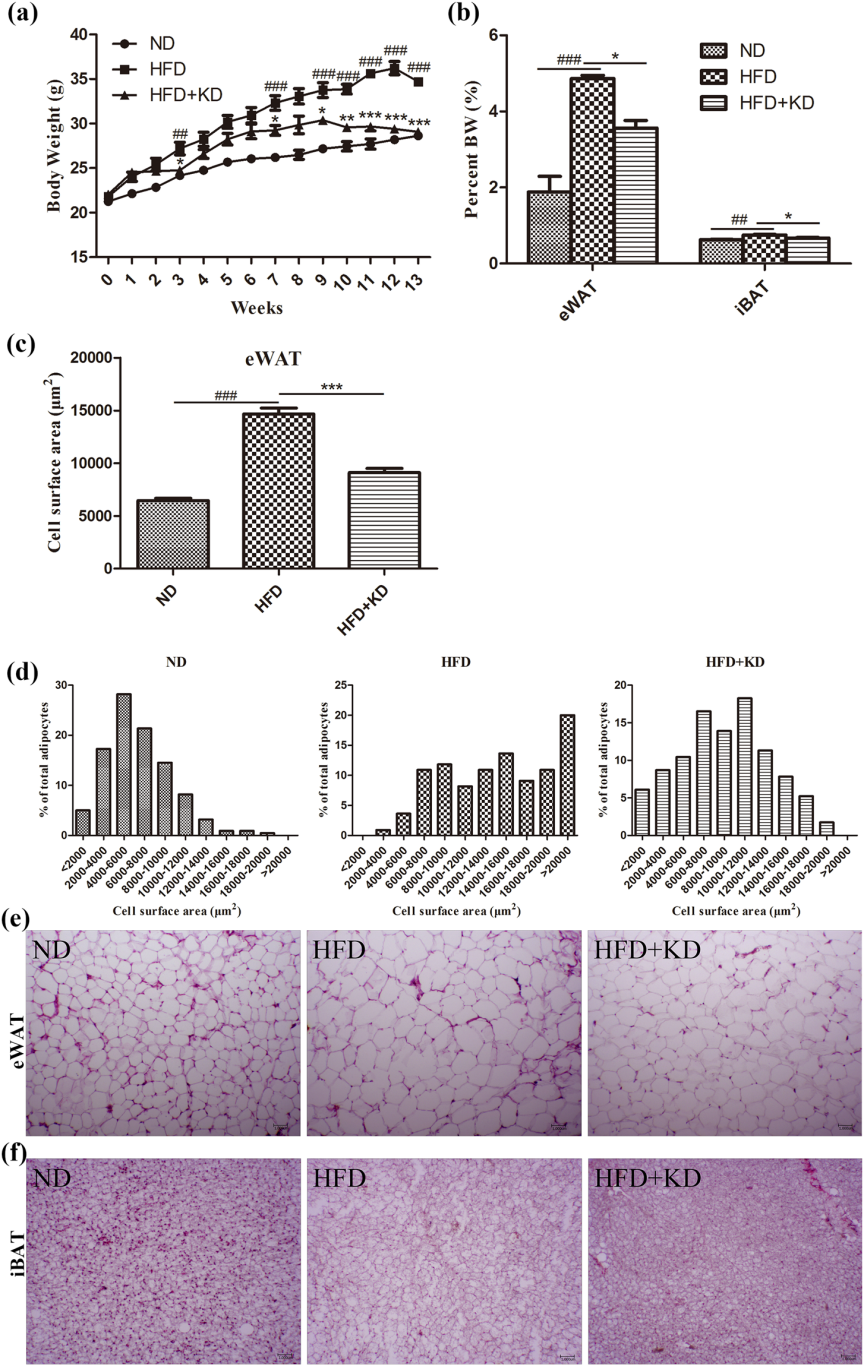
Body weight. Mice were fed ND(normal diet), HFD, or HFD + KD (0.33%) for 14 weeks. (**a**) Body weight. (**b**) The indices of eWAT and iBAT versus body weight (BW). (**c**) Cell surface area (CSA) of eWAT adipocytes. (**d**) Adipocyte size distribution of eWAT of all experimental groups. (**e** and **f**) Hematoxylin and eosin (H&E) staining of eWAT and iBAT adipocytes, magnification 100 × . n = 8 in each group. Significant differences between ND and HFD are indicated as ^###^P < 0.001; significant differences between HFD and HFD + KD are indicated as *P < 0.05, **P < 0.01, ***P < 0.001.

As described above, an aqueous extract of *Ilex latifolia* Thunb inhibited fatty acid accumulation in cells, raising the question of whether *Ilex latifolia* Thunb can prevent the development of obesity in animal models. To address this question, mice were fed a high-fat diet (HFD) either with or without aqueous extract of *Ilex latifolia* Thunb supplement. After mice were fed a HFD for 14 weeks, we found that mice treated with different dosage of aqueous extract of *Ilex latifolia* Thunb displayed similarly significantly reduced body weight gain compared to mice without treatment with *Ilex latifolia* Thunb (Figs 2a and S3). So middle-dose aqueous extract of *Ilex latifolia* Thunb was used to perform further experiments in this research. Mice treated with *Ilex latifolia* Thunb had significantly lower indices of epididymal white adipose tissue (eWAT) and intrascapular brown adipose tissue (iBAT) versus body weight than mice without *Ilex latifolia* Thunb, indicating a protective role of *Ilex latifolia* Thunb in the development of obesity (Fig. 2b). In the HFD group, mice fed *Ilex latifolia* Thunb had a smaller white and brown adipocyte size than controls, suggesting the inhibition of fatty acid accumulation (Fig. 2c–f). These observations demonstrate that aqueous extract of *Ilex latifolia* Thunb protected mice from fatty acid accumulation and reduced the body weight gain of the mice in the HFD group.

### *Ilex latifolia* Thunb enhances energy expenditure

**Figure 3:**
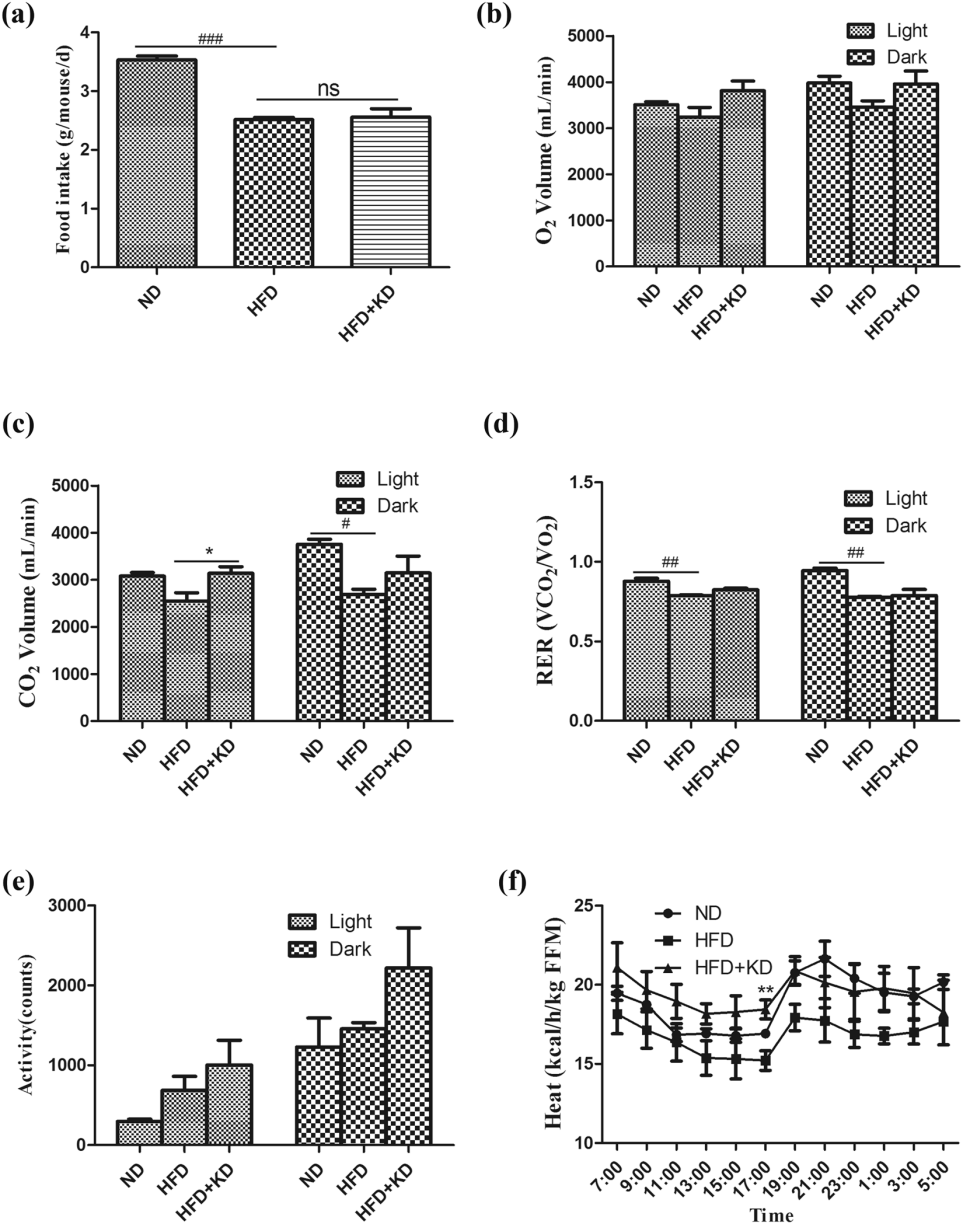
Energy balance. (**a**) Food intake (n = 8). (**b–f**) Metabolic chambers analysis of O_2_, CO_2_, RER, spontaneous physical activity and heat (n = 4). FFM: fat free mass. Significant differences between ND and HFD are indicated as ^###^P < 0.001; significant differences between HFD and HFD + KD are indicated as *P < 0.05, **P < 0.01.

To investigate the energy balance, we monitored food intake amounts and found no change in food consumption in mice treated with *Ilex latifolia* Thunb compared to mice without *Ilex latifolia* Thunb when fed a HFD, indicating that neither food intake nor the appetites of the mice were modulated by *Ilex latifolia* Thunb (Fig. 3a). After the mice were HFD-fed for 12 weeks, mice treated with *Ilex latifolia* Thunb showed more CO_2_ release than did controls (Fig. 3b–d). And heat productions were increased in mice treated with *Ilex latifolia* Thunb compared to mice without *Ilex latifolia* Thunb on a HFD (Fig 3e, f), supporting denser iBAT in Fig. 2f. These data suggest that *Ilex latifolia* Thunb attenuated HFD-induced obesity due to enhanced energy expenditure.

### *Ilex latifolia* Thunb protects against hepatic steatosis

**Figure 4:**
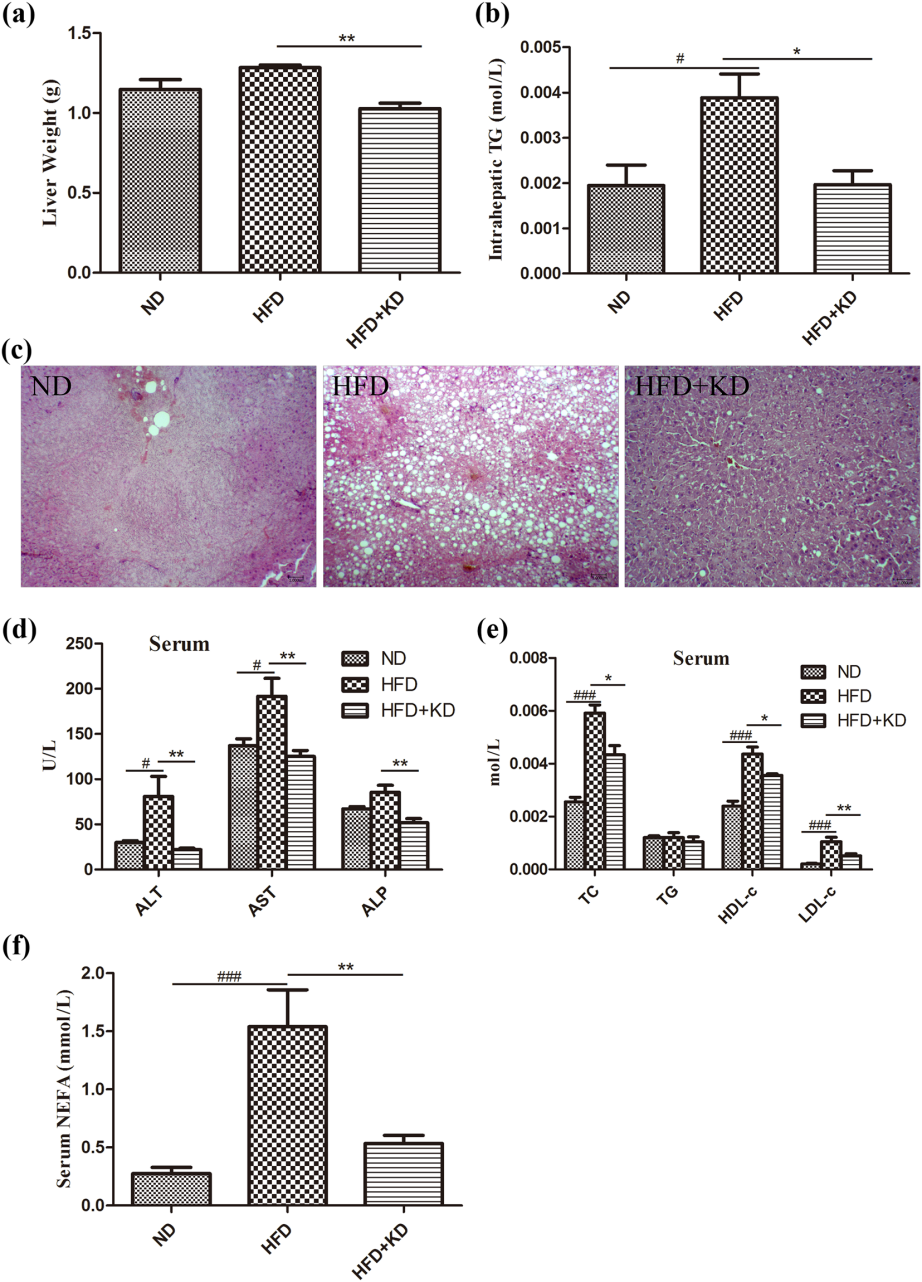
Liver function and serum lipid levels. (**a**) Weight of the liver. (**b**) Intrahepatic concentrations of TG. (**c**) H&E staining (×100) of liver sections. (**d**) The quantitative results of ALT, AST and ALP contents in serum. (**e**) Serum TC, TG, HDL-c and LDL-c levels were measured. (**f**) NEFA level in serum was measured. n = 8 for each group. Significant differences between ND and HFD are indicated as ^###^P < 0.001; significant differences between HFD and HFD + KD are indicated as *P < 0.05, **P < 0.01.

After being placed on a HFD for 14 weeks, mice treated with *Ilex latifolia* Thunb showed significantly lower liver weight than mice fed a HFD without *Ilex latifolia* Thunb (Fig. 4a). The analysis of intrahepatic triglyceride (TG) content revealed elevated TG levels in the livers of mice fed a HFD, which was prevented by *Ilex latifolia* Thunb treatment (Fig. 4b). Notably, we found that mice on a HFD showed macrovesicular steatosis, but mice treated with *Ilex latifolia* Thunb displayed markedly fewer fat droplets in the liver than the controls on a HFD, as revealed by hematoxylin and eosin (H&E) staining, indicating an inhibitory role of *Ilex latifolia* Thunb in hepatic steatosis (Fig. 4c). *Ilex latifolia* Thunb decreased the elevated alanine aminotransferase (ALT), aspartate aminotransferase (AST) and alkaline phosphatase (ALP) levels in mice fed a HFD compared to controls, suggesting a protective role of *Ilex latifolia* Thunb in hepatic injury (Fig. 4d). Moreover, mice treated with *Ilex latifolia* Thunb had significantly lower serum total cholesterol (TC), high-density lipoprotein cholesterol (HDL-c) and low-density lipoprotein cholesterol (LDL-c) levels than mice fed a HFD (Fig. 4e). However, the serum TG level was similar between mice fed a HFD plus *Ilex latifolia* Thunb and controls fed a HFD. In contrast, the HFD group showed a significantly higher level of serum non-esterified fatty acids (NEFA) compared with the normal diet (ND) group, and *Ilex latifolia* Thunb reduced the level of NEFA in circulation (Fig. 4f). Thus, these results demonstrate that an aqueous extract of *Ilex latifolia* Thunb prevents HFD-induced hepatic steatosis.

### *Ilex latifolia* Thunb improves insulin sensitivity

**Figure 5:**
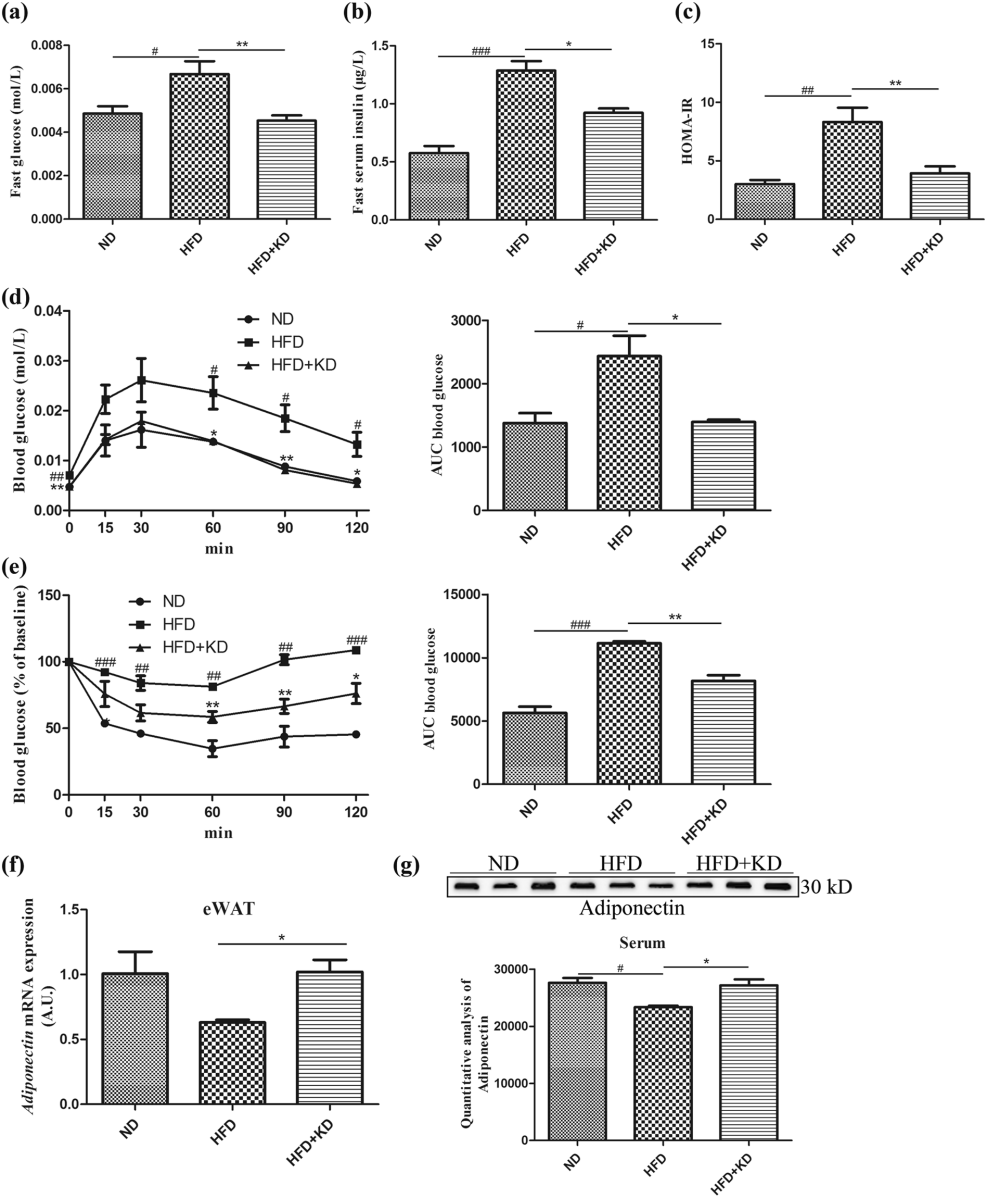
Insulin sensitivity. (**a**) Fasting blood glucose (n = 8). (**b**) Fasting insulin levels on ND, HFD or HFD + KD. (**c**): HOMA-IR. (**d**) Intraperitoneal GTT from mice fed each diet (left) and area under curve (AUC). (**e**) ITT for mice fed ND, HFD or HDF + KD (left) and area under curve (AUC). (**f**) Real-time qPCR results of gene expression levels of *Adiponectin* in eWAT. (**g**) Immunoblotting analysis of Adiponectin signaling in serum. Full-length blots are presented in Supplementary Figure S7. For b-g, n = 5 in each group. Significant differences between ND and HFD are indicated as ^###^P < 0.001; significant differences between HFD and HFD + KD are indicated as *P < 0.05, **P < 0.01.

The beneficial effects of *Ilex latifolia* Thunb on the development of obesity prompted us to investigate whether insulin sensitivity and glucose homeostasis of mice were affected by *Ilex latifolia* Thunb supplementation. As shown in Fig. 5a and b, fasting glucose and blood insulin levels were similarly elevated in mice fed a HFD compared to mice fed a ND, indicating the status of obesity-related hyperglycemia and hyperinsulinemia. In contrast, mice fed a HFD with *Ilex latifolia* Thunb exhibited normal blood insulin and glucose concentrations, suggesting the improvement of obesity-related insulin sensitivity and glucose homeostasis. Indeed, the HOMA-IR was significantly decreased in mice fed a HFD with *Ilex latifolia* Thunb compared to controls fed a HFD (Fig. 5c). To verify this improvement, we performed glucose tolerance tests (GTTs) and insulin tolerance tests (ITTs) on mice after they were fed on a HFD for 12 weeks (Fig. 5d and e). The mice fed a HFD showed a decreased response to glucose load, indicating the status of HFD-induced glucose intolerance. In contrast, mice fed a HFD with *Ilex latifolia* Thunb showed normal glucose tolerance levels compared to controls fed a ND, suggesting improved glucose homeostasis. Mice on a ND were more insulin sensitive than mice fed a HFD, indicating the development of obesity-related insulin resistance. The insulin sensitivity was similar between mice fed a HFD with *Ilex latifolia* Thunb and controls fed a ND, indicating that *Ilex latifolia* Thunb enhanced insulin sensitivity. Consistent with reports that adiponectin expression is associated with insulin sensitivity^15^, the mRNA level of *Adiponectin* was dramatically elevated in eWAT in mice fed a HFD supplemented with *Ilex latifolia* Thunb compared to that in HFD-control mice (Fig. 5f). The serum level of Adiponectin was also significantly elevated in mice treated with *Ilex latifolia* Thunb (Fig. 5g). Furthermore, the phosphorylation levels of Akt were increased in both the livers and the eWAT of *Ilex latifolia* Thunb-treated mice, as well as IRS1 protein level in the eWAT (Fig. S4). These results described above suggest that aqueous extract of *Ilex latifolia* Thunb protects mice from obesity-related insulin resistance.

### *Ilex latifolia* Thunb decreases the expression of lipogenic genes in liver and adipose tissue

**Figure 6:**
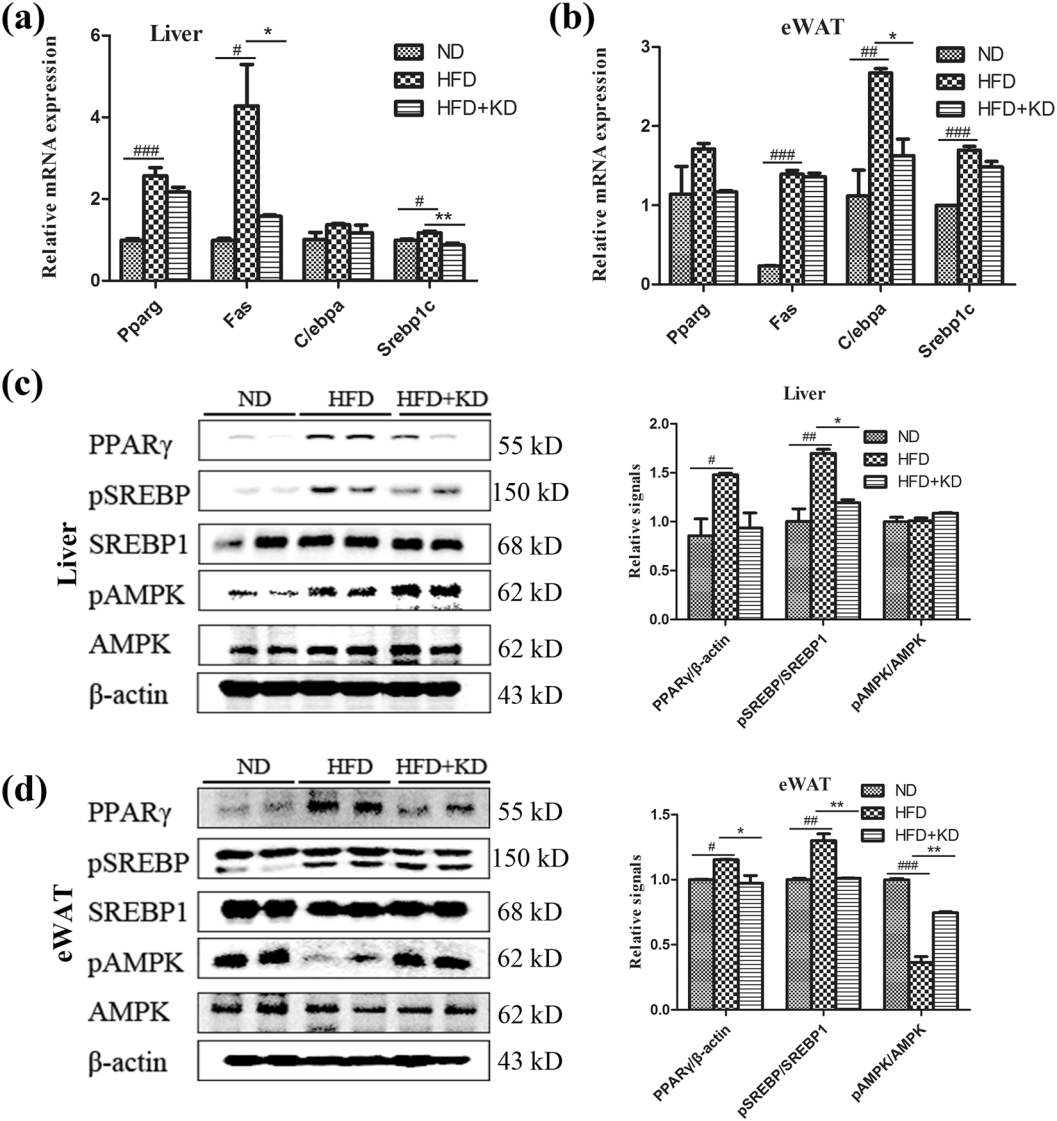
The expression of lipogenic genes in liver and adipose tissues. (**a**) mRNA expression levels of *Pparg(Ppar*_*γ*_ *)*, *Fas*, *C/ebpα* and *Srebp1c* in liver. (**b**) mRNA expression levels of *Pparg*, *Fas*, *C/ebpα* and *Srebp1c* in eWAT. (**c**) PPARγ, p-SREBP1c, SREBP-1c, p-AMPK and AMPK levels in liver. (**d**) PPARγ, p-SREBP1c, SREBP-1c, p-AMPK and AMPK levels in eWAT. Full-length blots are presented in Supplementary Figure S7. n = 5 for each group. Significant differences between ND and HFD are indicated as ^###^P < 0.001; significant differences between HFD and HFD + KD are indicated as *P < 0.05, **P < 0.01.

To probe the underlying molecular mechanism for the protective effects of *Ilex latifolia* Thunb on the development of obesity, we investigated the expression of lipogenic genes in both liver and adipose tissues. Quantitative PCR analysis showed that the mRNA levels of *Ppar*_*γ*_, *Fas* and *Srebp1c* in the liver were reduced in *Ilex latifolia* Thunb-treated mice compared with those in control mice, indicating an inhibitory effect of *Ilex latifolia* Thunb in liver lipogenesis (Fig. 6a). Indeed, immunoblotting results showed that PPARγ protein level and phosphorylation level of SREBP-1c were significantly enhanced in the liver of mice fed a HFD, compared to mice fed a ND, supporting the development of hepatic steatosis in Fig. 4c (Fig. 6c). Mice fed a HFD with *Ilex latifolia* Thunb exhibited lower levels of PPARγ protein and SREBP-1c phosphorylation in liver than control mice fed a HFD, indicating that *Ilex latifolia* Thunb inhibited the lipogenic signaling in liver. Consistent with the liver data, the mRNA levels of *C/ebpα* were decreased in the eWAT of mice fed a HFD with *Ilex latifolia* Thunb, suggesting that *Ilex latifolia* Thunb inhibited lipogenesis in adipose tissue (Fig. 6b). Indeed, significantly reduced PPARγ protein and phosphorylated-SREBP-1c levels in eWAT were observed in mice fed a HFD with *Ilex latifolia* Thunb (Fig. 6d). Meanwhile, the mRNA expression of lipolytic genes *Atgl*, *Hsl*, *Plin1* and *Adrb3* were elevated in the eWAT of HFD mice, suggesting a positive feedback regulation in lipolytic states (Fig. S5)^16,17^. Moreover, under the HFD condition, mice showed significantly higher phosphorylated-AMPK level in liver tissue than did controls. *Ilex latifolia* Thunb also increased the level of phosphorylated-AMPK in liver tissue, supporting the increased activity in Figure 3e ^18^. Notably, a significantly reduced phosphorylated-AMPK level was observed in the eWAT of mice fed a HFD compared to controls. However, *Ilex latifolia* Thunb supplement restored the phosphorylated-AMPK in eWAT to control levels, suggesting enhanced energy expenditure in those mice (Fig. 3f). Thus, these data suggest that *Ilex latifolia* Thunb protects mice from obesity by reducing lipogenesis.

### *Ilex latifolia* Thunb decreases inflammation

**Figure 7:**
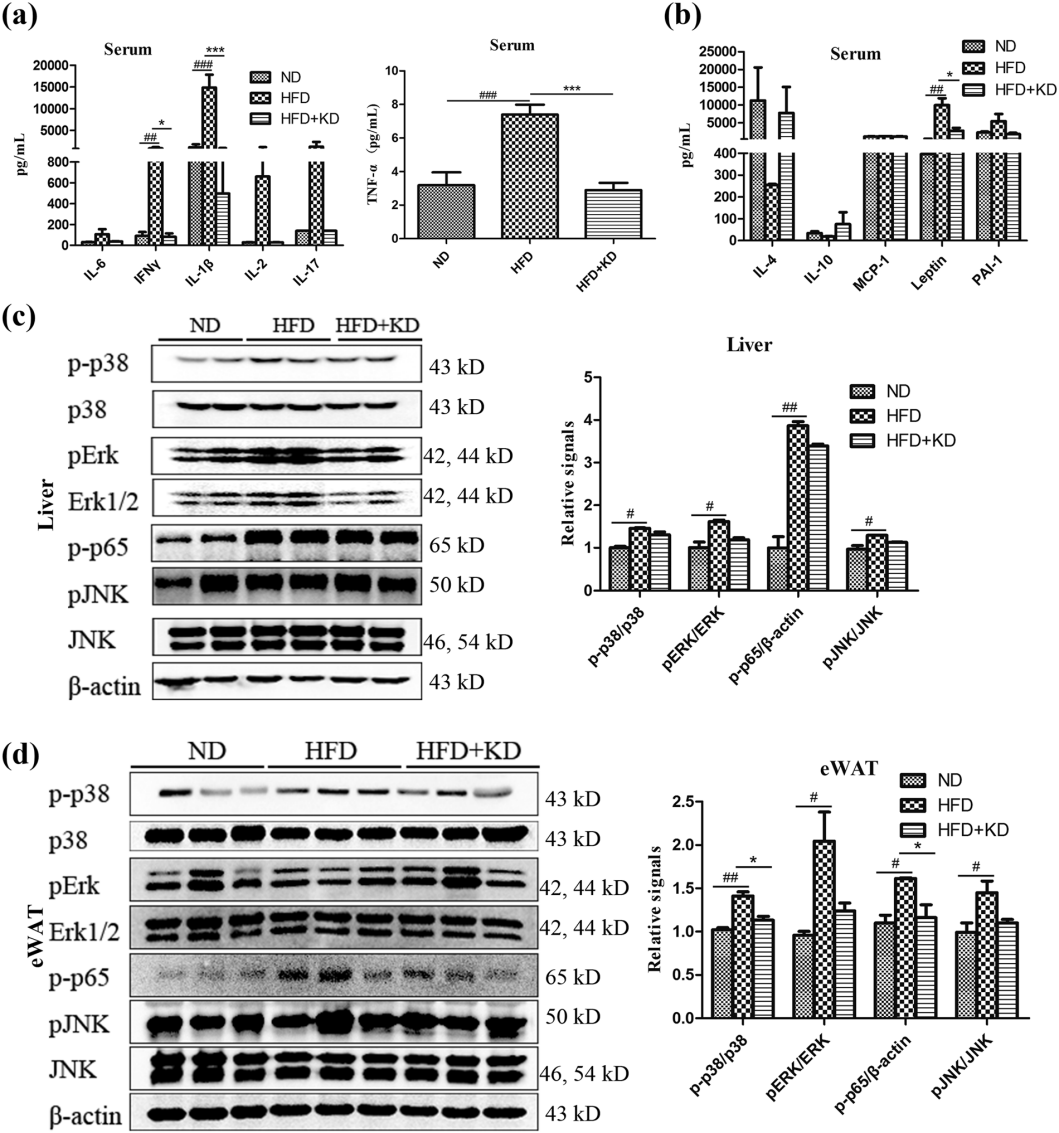
Inflammatory cytokine levels and cell signaling. (**a** and **b**) Cytokine levels in serum. (**c**) Immunoblotting analysis of p38 MAPK, Erk1/2, NF-κB p-p65 and JNK signaling in liver. (**d**) Immunoblotting analysis of p38 MAPK, Erk1/2, NF-κB p-p65 and JNK signaling in eWAT. Full-length blots are presented in Supplementary Figures S8 and S9. For a and b, n = 8 in each group, for c and d, n = 5 per group. Significant differences between ND and HFD are indicated as ^##^P < 0.01; significant differences between HFD and HFD + KD are indicated as *P < 0.05, ***P < 0.001.

Obesity is often associated with an inflammatory state in metabolic tissues and is defined as low-grade and chronic inflammation in response to excess nutrients and energy^19^. The inflammation in metabolic tissues including adipose and liver leads to a state of insulin insensitivity and metabolic dysfunction. Since aqueous extract of *Ilex latifolia* Thunb improved insulin sensitivity and ameliorated metabolic disorders, we next examined whether *Ilex latifolia* Thunb could prevent low-grade inflammation in mice under HFD conditions. Mice fed a HFD showed higher serum pro-inflammatory cytokine levels of IL-6, TNF-alpha, IFN-gamma, IL-1β, IL-2 and IL-17 and lower serum anti-inflammatory cytokine levels of IL-4 and IL-10 than those of mice fed a ND, suggesting an inflammatory status. Remarkably, under the HFD condition, pro-inflammatory cytokine levels of IL-6, TNF-alpha, IFN-gamma, IL-1β, IL-2 and IL-17 and anti-inflammatory cytokine concentrations of IL-4 and IL-10 were restored to control levels in mice treated with *Ilex latifolia* Thunb, indicating that *Ilex latifolia* Thunb reduces pro-inflammatory markers in the circulation (Fig. 7a, b). Consistently, phosphorylated p38 MAPK and p-p65 (p65 constitutes a subunit of NF-κB) levels in eWAT were significantly increased in mice fed a HFD compared to controls fed a ND, and they were dramatically decreased by *Ilex latifolia* Thunb, whereas the activation of pErk1/2 and pJNK in both liver and eWAT were similar between *Ilex latifolia* Thunb-treated mice and controls, indicating *Ilex latifolia* Thunb decreased activity of pro-inflammatory signaling pathways such as p38 MAPK and p65 NF-κB in eWAT (Fig. 7c and d). Moreover, the aqueous extract of *Ilex latifolia* Thunb decreased mRNA levels of *Il6* and *Mcp1* in the eWAT in HFD + KD mice compared to HFD fed mice (Fig. S6). In addition to inflammatory cytokines, the levels of the adipokine leptin were also elevated in mice fed a HFD compared to controls, and *Ilex latifolia* Thunb supplement reversed the high levels of leptin to normal concentrations, suggesting a regulatory role of *Ilex latifolia* Thunb in adipokine production (Fig. 7b). Together, these observations reveal that *Ilex latifolia* Thunb reduces HFD-induced inflammation via the modulation of cytokine levels and via p38 MAPK and p65 NF-κB signaling.

## Discussion

In this study, we provide the evidence that an aqueous extract of *Ilex latifolia* Thunb prevents HFD-induced body weight gain. The aqueous extract of *Ilex latifolia* Thunb decreases gain in body weight as well as the indices of eWAT and iBAT versus body weight, and it improves liver function, obesity-related insulin resistance and chronic inflammation in HFD-fed mice. Lipid levels in liver and blood glucose of mice were significantly reduced, and the expression of lipogenic genes was dramatically altered by *Ilex latifolia* Thunb treatment. These results suggest that an aqueous extract of *Ilex latifolia* Thunb may play a protective role in obesity, T2D, non-alcoholic fatty liver disease (NAFLD) and chronic inflammation.

Previous studies have shown that the ethanolic extract but not the aqueous extract of *Ilex kudingcha* C.J. Tseng inhibited adipocyte differentiation and the development of obesity, suggesting that liposoluble components of *Ilex kudingcha* C.J. Tseng, including lupeol and ursolic acid, may act as the anti-adipogenic and anti-metabolic syndrome active compound candidates^1^. In contrast, the aqueous extract of *Ilex kudingcha* C.J. Tseng was found to possess an anti-diabetic activity, and chlorogenic acids, dicaffeoylquinic acids, flavonoids, triterpenoid saponins and other natural products were speculated as being active compound candidates^7,11^. Therefore, the truly active compounds from *Ilex kudingcha* C.J. Tseng are still unknown. Here, the anti-obesity active components present in *Ilex latifolia* Thunb were water soluble, and their role in lipogenesis is totally different from the aqueous and ethanolic extracts of *Ilex kudingcha* C.J. Tseng, suggesting different active components in the aqueous extracts of *Ilex latifolia* Thunb and *Ilex kudingcha* C.J. Tseng. However, further experiments are required for the identification of the active components of *Ilex latifolia* Thunb that have an anti-obesity role.

Kuding tea is a traditional beverage in China. Like the popular green tea, kuding tea is used in health care formulas to ameliorate metabolic disorders such as obesity. The large-leaved kuding tea has exhibited many biological functions on health^6^. Kuding tea *Ilex kudingcha* C.J. Tseng is able to lower the levels of serum TC, LDL-c, and HDL-c in mice, suppress rat liver mitochondrial peroxidation and prevent the progression of atherosclerosis^20,21^. Similarly, in the current study, body weight and serum TC, LDL-c, HDL-c, ALT, AST, ALP and glucose levels were significantly increased in mice fed a HFD for 14 weeks. The aqueous extract of *Ilex latifolia* Thunb treatment reversed body weight gain, serum levels of TC, LDL-c, HDL-c, ALT, AST, and ALP, and glucose tolerance and insulin sensitivity in mice, suggesting that *Ilex latifolia* Thunb prevents the development of metabolic syndrome. However, more detailed analysis is required to dissect the molecular mechanism of the protective role of *Ilex latifolia* Thunb in dyslipidemia.

Although reduction of food intake may cause body weight loss and reduce the levels of blood glucose and lipids, we did not observe a difference in food intake between mice fed a HFD and mice fed a HFD with the aqueous extract of *Ilex latifolia* Thunb, suggesting no change in appetite. However, CO_2_ release and heat production were increased in mice treated with *Ilex latifolia* Thunb compared to controls. Therefore, the protective roles of the aqueous extract of *Ilex latifolia* Thunb against body weight gain may be associated with enhanced energy expenditure but independent of food intake or appetite. Consistently, the activation of AMPK, an energy expenditure marker, was significantly increased in adipose tissue by *Ilex latifolia* Thunb supplement. Additionally, adipocyte differentiation plays a key role in the pathogenesis of obesity^22^. Weight gain is a consequence of the increase in adipocyte volume (hypertrophy) and numbers (hyperplasia) caused by excess calories stored as TG, while weight loss was usually caused by a reduction of the adipocyte mass and number by suppression of energy intake or overburn of excess calories^23,24^. Here, the anti-adipogenesis and anti-obesity effects of *Ilex latifolia* Thunb were further examined in OP9 mouse stromal cells. The *Ilex latifolia* Thunb treatment inhibited the adipocyte differentiation and accumulation of fatty acids in OP9 mouse stromal cells, suggesting an inhibitory effect of *Ilex latifolia* Thunb in lipogenesis. Indeed, mice treated with *Ilex latifolia* Thunb have smaller adipocytes and fewer fat droplets in the liver than controls fed a HFD. However, markers for adipogenesis C*ebpα* and C*ebpβ* gene expression is not changed in OP9 cells and tissue from mice treated with the extract of *Ilex latifolia* Thunb, indicating complicated effects of the extract of *Ilex latifolia* Thunb on lipogenesis. Further experiments are required to determine how *Ilex latifolia* Thunb inhibits lipogenesis and increases energy expenditure.

Low-grade inflammation is closely related with HFD-induced obesity. A HFD induces the expression of several pro-inflammatory cytokines (TNF-α, NF-κB, IL-6, IL-1β and MCP-1) as well as the activation of JNK (c-Jun N-terminal kinase) in white adipose tissue and liver^25^. In our study, similar inflammatory responses were observed in HFD-fed mice compared to ND controls. As a pure natural health herb, *Ilex latifolia* Thunb suppressed HFD-induced chronic inflammation, as evidenced by a modulated cytokine profile compared with HFD models, although a more detailed analysis is required to elucidate the molecular mechanism. These findings suggest that the aqueous extract of *Ilex latifolia* Thunb may be used as a potential dietary strategy for preventing metabolic disorders such as obesity, diabetes, non-alcoholic steatohepatitis and atherosclerosis. The potential of using naturally occurring dietary supplements to regulate body weight and lipid metabolism is attractive. Because this traditional beverage is safe and inexpensive, it should be considered as a dietary therapy for metabolic syndrome. This type of therapy is particularly important because weight loss and existing treatments for NAFLD have poor long-term success rates. Further investigations are required to identify the active compounds in *Ilex latifolia* Thunb, their roles in metabolic diseases, and the molecular mechanisms by which this supplement protects against obesity.

## Materials and Methods

### Cell culture and differentiation

The leaves of *Ilex latifolia* Thunb were from Hainan. Rosiglitazone (R) was purchased from Sigma Ltd. (USA). *Ilex latifolia* Thunb with a material:liquid ratio of 1:10 were boiled in distilled water for 3 h. The extracts were concentrated on a rotary evaporator under reduced pressure. OP9 mouse stromal cells were obtained from the ATCC (USA) and maintained in MEM-α containing L-Glutamine, with 20% FBS (Secure fetal bovine serum; Gibco, Australia), 100 U/mL penicillin, and 100 μg/mL streptomycin^26^. For adipocyte differentiation, 1 μM rosiglitazone was added into the culture for differentiation at 37 °C in 5% CO_2_. Aqueous extract of *Ilex latifolia* Thunb was added into the medium at 4 μg/mL for 5 days. Three wells of OP9 cells were used for each experiment. After 5 days of treatment, the cells were washed twice with PBS, fixed with 4% paraformaldehyde at room temperature for 30 minutes and then stained with oil red O (Sigma, USA), and the absorbance was measured at 510 nm.

### Quantitative real-time PCR

Total RNA was extracted using TRIzol (Ambion, USA). cDNA was synthesized by a reverse transcription reagent kit (RT Reagent Kit with gDNA Eraser RR047A, TaKaRa, Japan). Gene expression levels were analyzed by quantitative real-time PCR using the BIO-RAD CFX Connect Real-Time System (CA, USA). The primers of *Pparγ* (NM_001127330.2): 5′-TCGCTGATGCACTGCCTATG-3, 5′-GAGAGGTCCACAGAGCTGATT-3′; *Fas* (NM_007988.3): 5′-GGAGGTGGTGATAGCCGGTAT-3′, 5′-TGGGTAATCCATAGAGCCCAG-3′; *C/ebpα* (NM_001287514.1): 5′-CAAGAACAGCAACGAGTACCG-3′, 5′-GTCACTGGTCAACTCCAGCAC-3′; *C/ebpβ* (NM_001287738.1): 5′-CGACTTCAGCGCCTACATTGA-3′, 5′-CTAGCGACAGACCCCACAC-3′; *Srebp1c* (NM_001313979.1): 5′-GAGCGAGCGTTGAACTGTAT-3′, 5′-ATGCTGGAGCTGACAGAGAA-3′; *Adiponectin* (NM_009605.5): 5′-TGTTCCTCTTAATCCTGCCCA-3′, 5′-CCAACCTGCACAAGTTCCCTT-3′; *Il6* (NM_001314054.1): 5′-CCAAGAGGTGAGTGCTTCCC-3′, 5′-CTGTTGTTCAGACTCTCTCCCT-3′; *Mcp1* (NM_011333.3): 5′-TCACCTGCTGCTACTCATTC-3′, 5′-TACAGAAGTGCTTGAGGTGG-3′; *Atgl* (NM_001163689.1): 5′-CAACGCCACTCACATCTACGG-3′, 5′-GGACACCTCAATAATGTTGGCAC-3′; *Hsl* (NM_001039507.2): 5′-TCCCTCAGTATCTAGGCCAGA-3′, 5′-GGCTCATTTGGGAGACTTTGTTT-3′; *Plin1* (NM_001113471.1): 5′-GGGACCTGTGAGTGCTTCC-3′, 5′-GTATTGAAGAGCCGGGATCTTTT-3′; *Adrb3* (NM_013462.3): 5′-GGCCCTCTCTAGTTCCCAG-3′, 5′-TAGCCATCAAACCTGTTGAGC-3′; *β-actin* (NM_007393.5): 5′-GGCTGTATTCCCCTCCATCG-3′, 5′-CCAGTTGGTAACAATGCCATGT-3′ were used to perform qPCR.

### HFD feed and Metabolic assays

The animal protocols used in this study were approved by the Animal Ethics Committee of Jiangnan University, China. All experiments were performed in accordance with China regulations for the administration of affairs concerning experimental animals 2017. Male C57BL/6 mice (6 weeks old) were purchased from the Xi Nuo Sai BioScience, Inc. (Suzhou, China) and were randomly separated into three groups: normal diet (ND; chow diet, 10% of calories derived from fat); high-fat diet (HFD; 45% of calories derived from fat, Research Diets, Beijing, China; D12451); and HFD + KD (0.33% aqueous extract of *Ilex latifolia* Thunb was added to the HFD) after acclimatization for 1 week. The diet study was continued for 14 weeks. Hematoxylin and eosin (H&E) staining of epididymal white adipose tissue (eWAT), intrascapular brown adipose tissue (iBAT) and liver were performed as in previous reports^27,28^. The eWAT adipocyte cell surface area (CSA) from at least 150 cells/section was measured using the software Adobe Photoshop CS5^29^. Intrahepatic triglyceride (TG) content was measured by colorimetric methods (Triglyceride Quantification Kit, Bio Vision, USA)^30^. Respiratory exchange ratio (RER, the volume ratio of CO_2_ exhaled versus O_2_ consumed), heat (kcal/h/kg FFM) and spontaneous locomotor activity (X Ambulatory counts) were measured using metabolic chambers (Columbus Instruments, Columbus, OH). After 14 weeks of HFD feeding, the mice were sacrificed, and serum was collected. Serum TG, TC, HDL-c, LDL-c, ALT, AST and ALP levels were measured using Roche Modular P800 Automatic Analyzer (Roche, Basel, Switzerland). Glucose tolerance tests (GTTs) and insulin tolerance tests (ITTs) were performed at the end of this experiment as in previous reports^31^. For the GTT, mice were fasted overnight for 12 hours, and glucose (2 g/kg body weight) was injected intraperitoneally. For the ITT, insulin (0.75 U/kg body weight) was injected intraperitoneally. The blood samples were collected for the determination of glucose values with an Accu-chek glucometer (Roche Diagnostics, Basel, Switzerland). Insulin levels in serum were measured using an ELISA kit (Mouse Insulin ELISA, Mercodia, Sweden) according to the standard procedure. The intra- and inter-assay coefficient of variation for the insulin ELISA kit are < 4% and < 6%, respectively. Mouse cytokines in serum were measured using Milliplex Map kits (EMD Millipore Corporation, USA) by Luminex (USA) and a TNF-α ELISA kit (BioLegend, USA). The intra- and inter-assay coefficient of variation for the Luminex assays are < 5% and < 10%. Levels of non-esterified fatty acids (NEFA) in serum were determined by an enzymatic kit (Nanjing Jiancheng Bioengineering Institute, China).

### Cell signaling

The tissues were lysed with RIPA buffer containing protease inhibitors (Sangon Biotech, Shanghai). Immunoblotting was performed as in previous reports^28,32^. Tissue lysates were immunoblotted for SREBP-1c (Santa Cruz Biotechnology), p-AMPK (Ser485, Santa Cruz Biotechnology), β-actin (Santa Cruz Biotechnology), PPARγ (Cell Signaling), p-SREBP1c (Ser372, Cell Signaling), AMPK (Cell Signaling), p-p38 (Thr180/Tyr182, Cell Signaling), p38 MAPK (Cell Signaling), p-Erk1/2 (Thr202/Tyr204, Cell Signaling), Erk1/2 (Cell Signaling), p-Akt (Ser 473, Cell Signaling), Akt (Cell Signaling), NF-κB p-p65 (Ser 536, Cell Signaling), p-JNK (Thr183/Tyr185, Cell Signaling), JNK (Cell Signaling), IRS1 (Cell Signaling) and Adiponectin (Abcam)^31,32^.

### Statistical analysis

Each experiment was repeated at least three times. All data are presented as the mean ± SEM. Data were analyzed by one-way ANOVA or two-way ANOVA. *P < 0.05; **P < 0.01; ***P < 0.001.

## Electronic supplementary material

Supplementary Information
