# Supplementary material for: Ilex latifolia Thunb protects mice from HFD-induced body weight gain
Source: Sci Rep. 2017 Nov 7;7:14660. doi: 10.1038/s41598-017-15292-x (PMC5676986; doi:10.1038/s41598-017-15292-x)
Supplement: Supplementary file 1 — Supplementary Information [file 41598_2017_15292_MOESM1_ESM.pdf]

Ilex latifolia Thunb protects mice from HFD-induced body weight gain

Hailan Wu[1,2#], Yue-Lei Chen[4#], Yueyuan Yu[1,2], Jin Zang[1,2], Yikuan Wu[1,2] and Zhao He[1,2,3]*

1. State Key Laboratory of Food Science and Technology, School of Food Science and Technology, Jiangnan University, Wuxi, 214122, China.
2. Synergistic Innovation Center for Food Safety and Nutrition, School of Food Science and Technology, Jiangnan University, 1800 Lihu Avenue, Wuxi, Jiangsu, 214122, China.
3. Institute of Endocrinology and metabolism, Shandong Academy of Clinical Medicine, Shandong Provincial Hospital affiliated to Shandong University, Jinan, Shandong, 250021, China.
4. Institute of Biochemistry and Cell Biology, Shanghai Institutes for Biological Sciences, Chinese Academy of Sciences, Shanghai, 200031, China.

# These authors contributed equally to this work.

Running title: *Ilex latifolia* Thunb prevents HFD-induced obesity

Zhao He, Ph.D.

School of Food Science and Technology

Jiangnan University

1800 Lihu Avenue

Wuxi, China 214122

Corresponding author: Zhao He (*zhaohe@jiangnan.edu.cn* or *87425764@qq.com*)

Supplementary Figure

Fig. S1, Wu et al

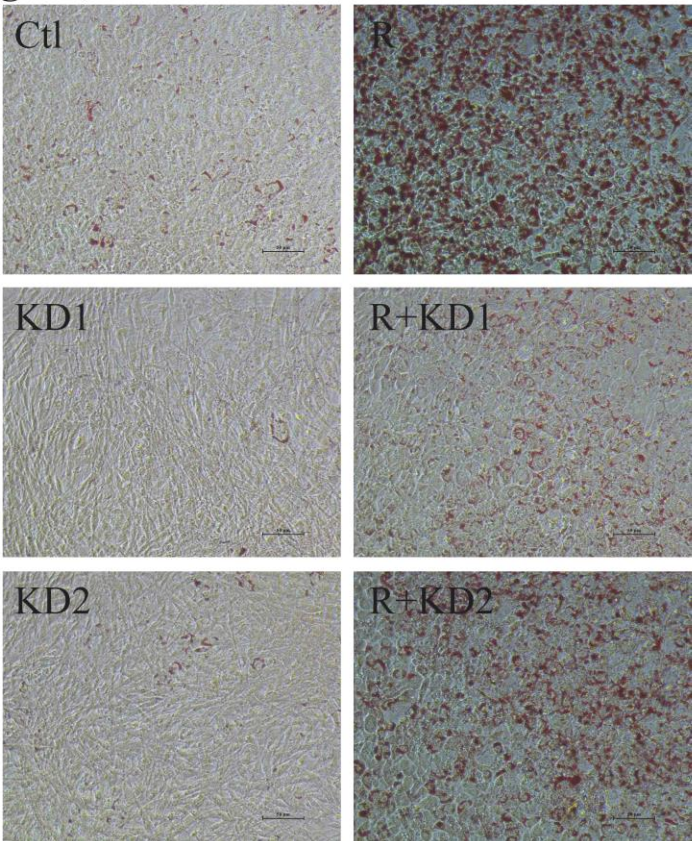

Figure S1. The oil red O staining of OP9 mouse stromal cells. The aqueous extract of *Ilex latifolia* Thunb and *Ilex kudingcha* C.J. Tseng were added into the medium at the concentration of 4 μg/mL. PBS was used as the vehicle control. Ctl: control; R: rosiglitazone; KD1: aqueous extract of *Ilex latifolia* Thunb; KD2: aqueous extract of *Ilex kudingcha* C.J. Tseng.

Fig. S2, Wu et al

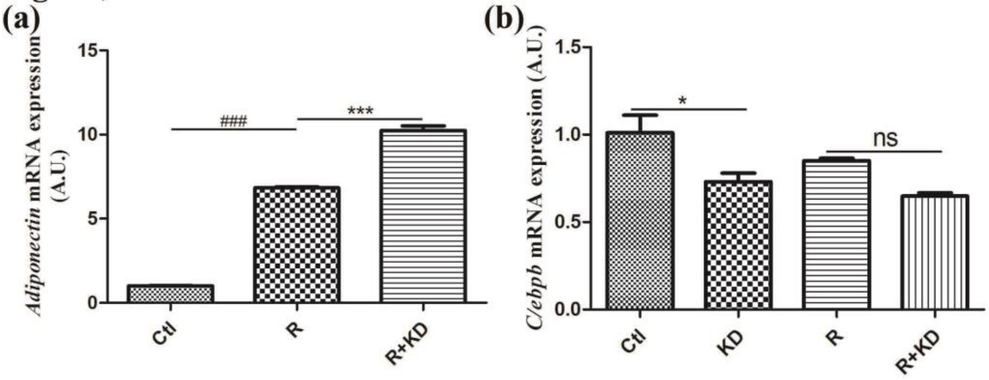

Figure S2. The mRNA levels of *Adiponectin* and *C/ebpb* in OP9 mouse stromal cells. Ctl: control; R: rosiglitazone; KD: aqueous extract of *Ilex latifolia* Thunb. *β-actin* was used as an internal control. Data are presented as mean ± SEM. Significant difference between Ctl and R are indicated as $^{###}$P<0.001; significant difference versus R+KD or KD are indicated as *P<0.05, ***P<0.001.

Fig. S3, Wu et al

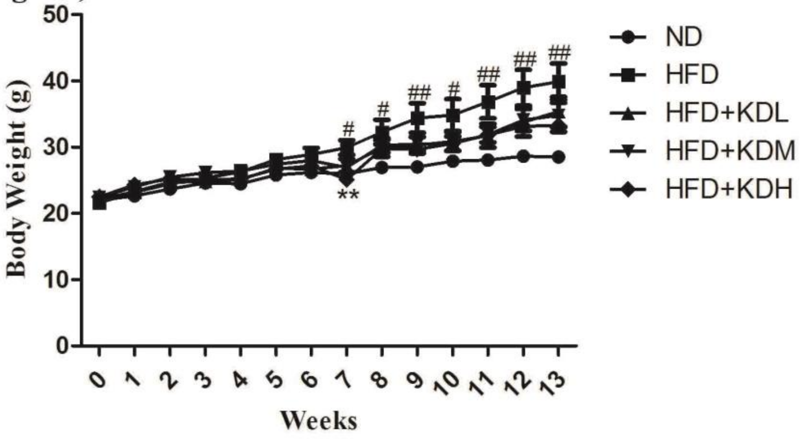

Figure S3. The dose study for the aqueous extract of *Ilex latifolia* Thunb. KDL: low-dose aqueous extract of *Ilex latifolia* Thunb (0.17%); KDM: middle-dose aqueous extract of *Ilex latifolia* Thunb (0.33%); KDH: high-dose aqueous extract of *Ilex latifolia* Thunb (0.66%). Data are presented as mean ± SEM. Significant difference between ND and HFD are indicated as $^{\#}P<0.05$; significant difference between HFD and HFD+KD are indicated as $^{**}P<0.01$.

Fig. S4, Wu et al

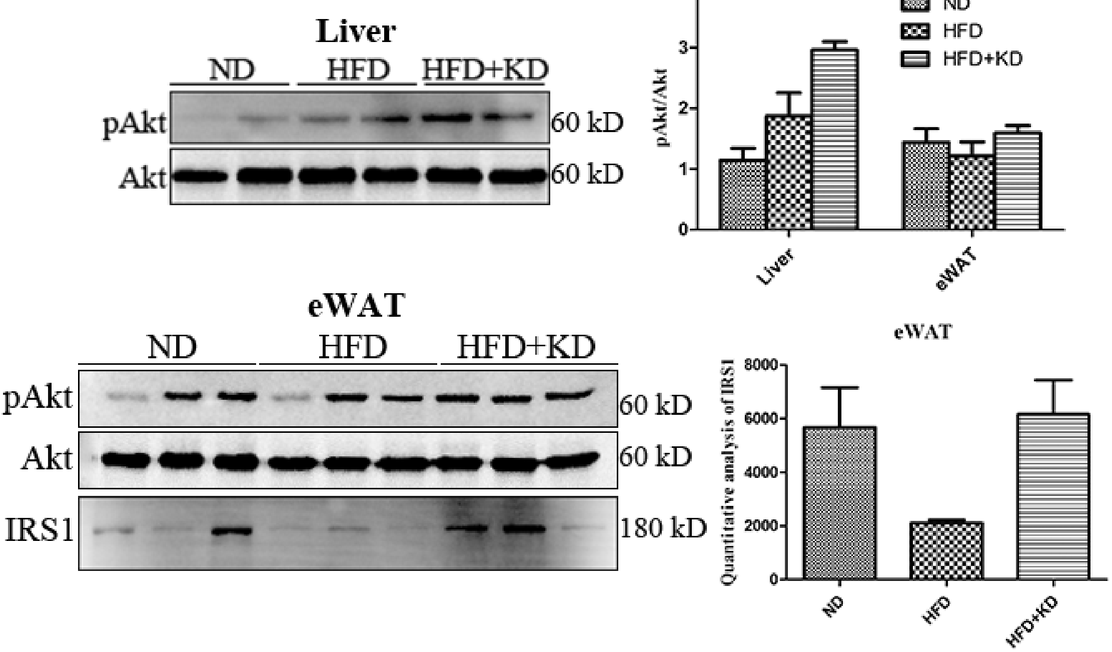

Figure S4. Immunoblotting analysis of Akt signaling in liver and eWAT, IRS1 signaling in eWAT.

Fig. S5, Wu et al

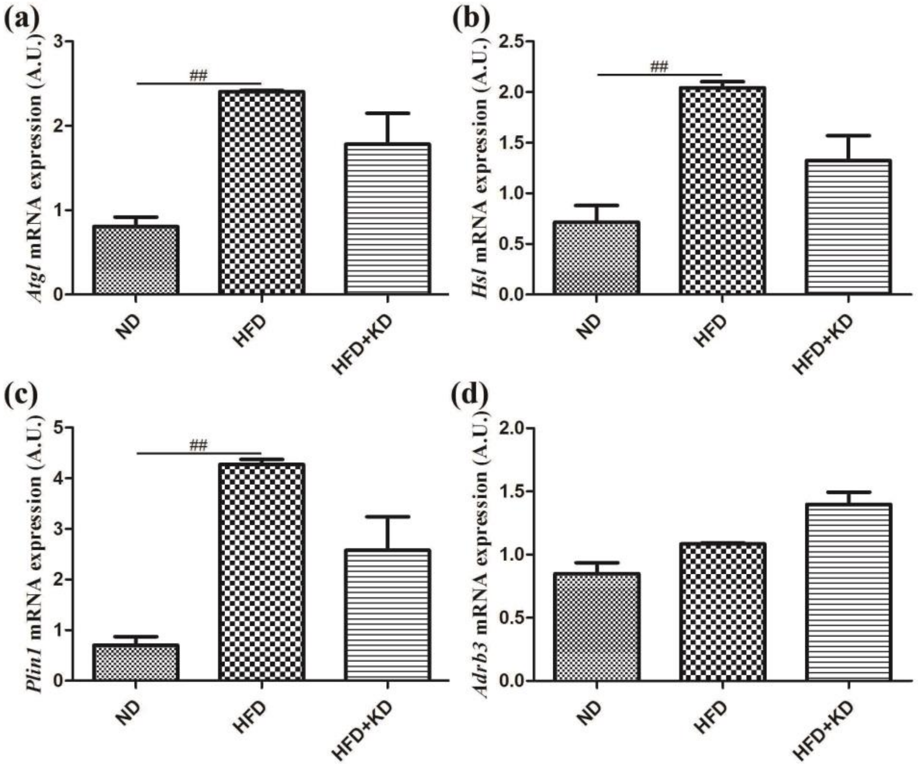

Figure S5. The expression of lipolytic genes in eWAT. Data are presented as mean ± SEM. Significant difference between ND and HFD are indicated as $^{\#\#}$P<0.01.

Fig. S6, Wu et al

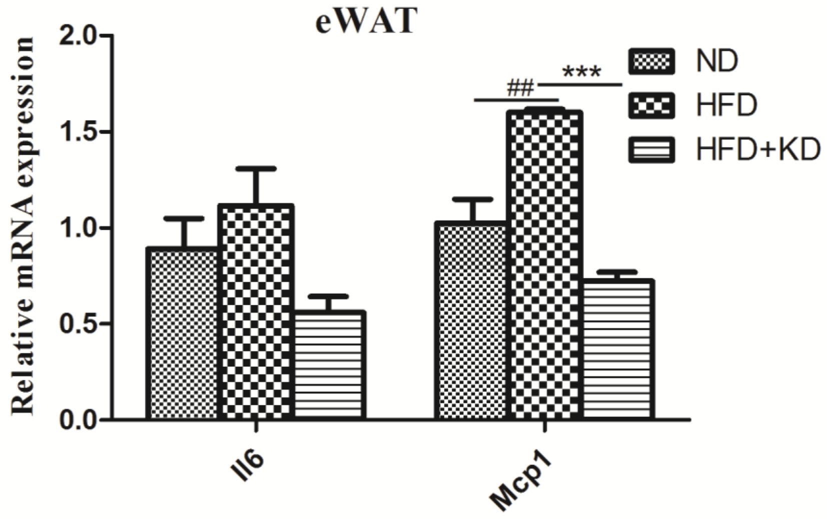

Figure S6. The expression of inflammatory genes in adipose tissues. mRNA expression levels of *Il6* and *Mcp1* in eWAT. Data are presented as mean ± SEM. Significant difference between ND and HFD are indicated as $^{\#\#}P<0.01$; significant difference between HFD and HFD+KD are indicated as $^{***}P<0.001$.

Fig. S7, Wu et al

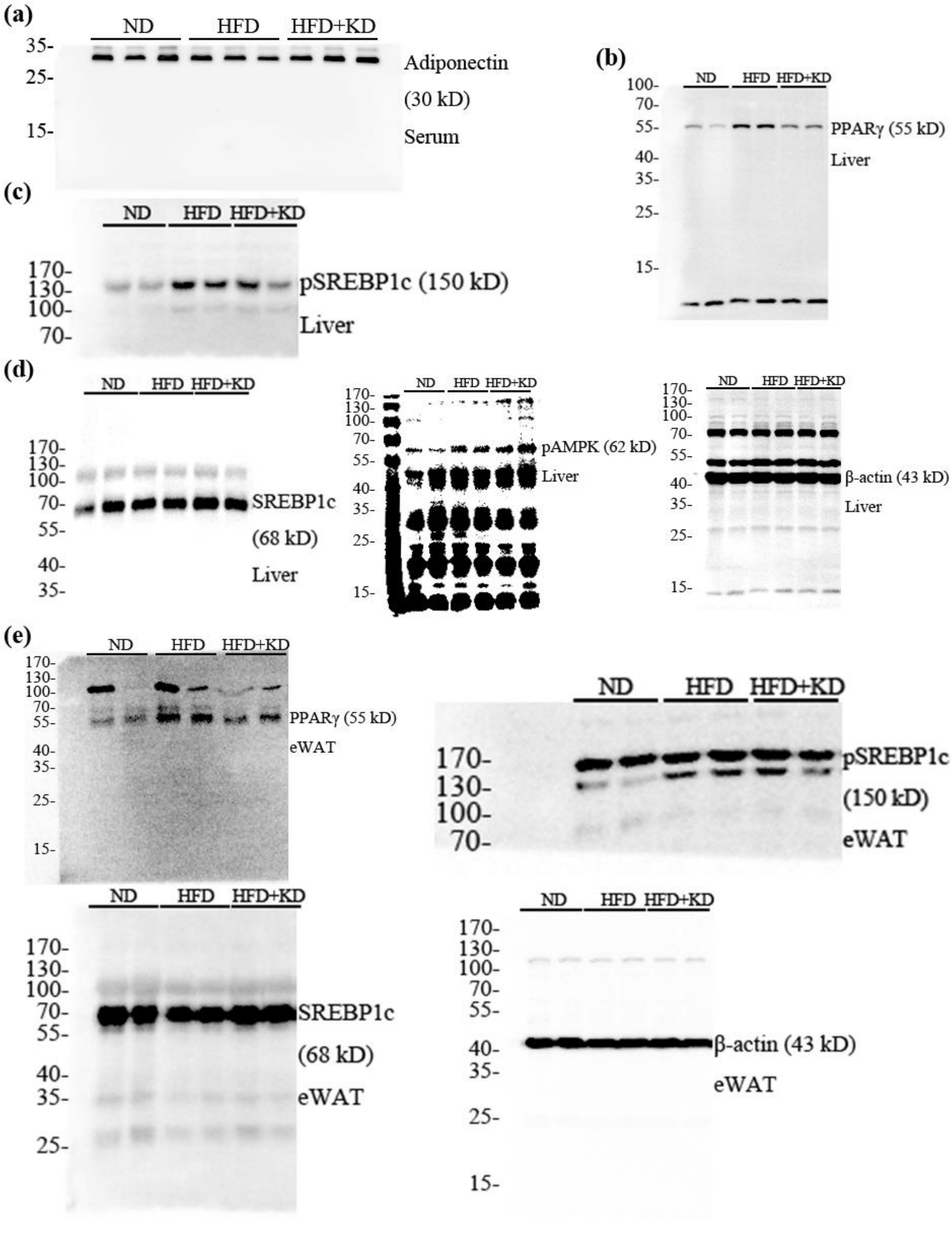

Figure S7. Scanned images of immunoblotting. Originals for cropped bands in: (a) Figure 5 panel g. (b-d) Figure 6 panel c. (e) Figure 6 panel d.

Fig. S8, Wu et al

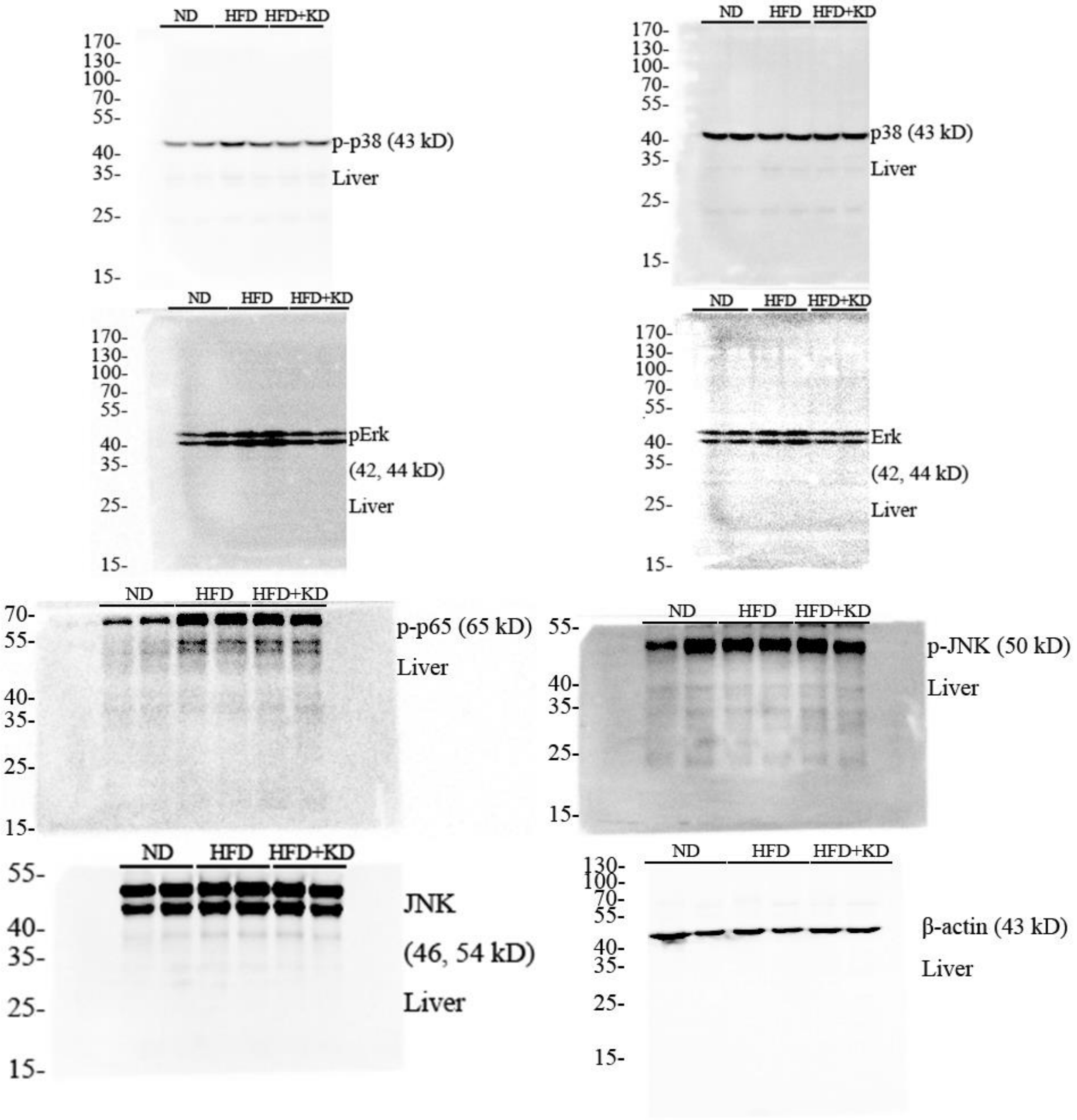

Figure S8. Scanned images of immunoblotting. Originals for cropped bands in Figure 7 panel c.

Fig. S9, Wu et al

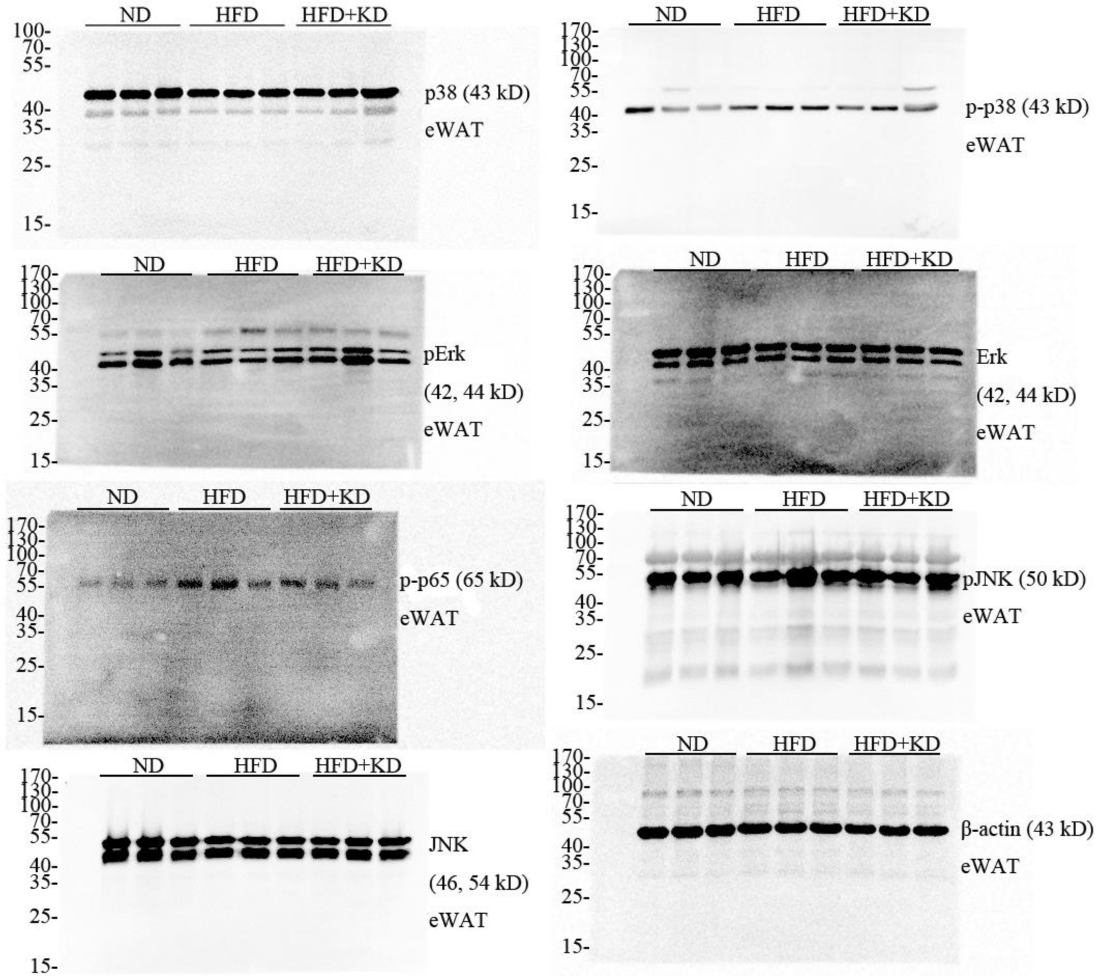

Figure S9. Scanned images of immunoblotting. Originals for cropped bands in Figure 7 panel d.
